# Intravenous infusion of bone marrow-derived mesenchymal stem cells improves tissue perfusion in a rat hindlimb ischemia model

**DOI:** 10.1038/s41598-022-18485-1

**Published:** 2022-10-10

**Authors:** Shusaku Maeda, Takuji Kawamura, Masanori Sasaki, Kazuo Shimamura, Takashi Shibuya, Akima Harada, Osamu Honmou, Yoshiki Sawa, Shigeru Miyagawa

**Affiliations:** 1 Department of Cardiovascular Surgery, Osaka University Graduate School of Medicine, 2-15 Yamadaoka, Suita, Osaka 565-0871 Japan; 2 Department of Neural Regenerative Medicine, Research Institute for Frontier Medicine, Sapporo Medical University School of Medicine, Sapporo, Hokkaido Japan

**Keywords:** Mesenchymal stem cells, Cardiovascular biology

## Abstract

Intravenous infusion of stem cells is a minimally invasive cellular delivery method, though a few have been reported in a critical limb-threatening ischemia (CLTI) animal model or patients. In the present study, we hypothesized that intravenous infusion of bone-marrow derived mesenchymal stem cells (MSCs) improves tissue perfusion in a rat hindlimb ischemia model. Hindlimb ischemia was generated in Sprague–Dawley rats by femoral artery removal, then seven days after ischemic induction intravenous infusion of 1 × 10^6^ MSCs (cell group) or vehicle (control group) was performed. As compared with the control, tissue perfusion was significantly increased in the cell group. Histological findings showed that capillary density was significantly increased in the cell group, with infused green fluorescent protein (GFP)-MSCs distributed in the ischemic limb. Furthermore, gene expression of vascular endothelial growth factor (VEGF) was significantly increased in ischemic hindlimb muscle tissues of rats treated with MSC infusion. In conclusion, intravenous infusion of bone-marrow derived MSCs improved tissue perfusion in ischemic hindlimbs through angiogenesis, suggesting that intravenous infusion of MSCs was a promising cell delivery method for treatment of CLTI.

## Introduction

Critical limb-threatening ischemia (CLTI) is a serious disorder and associated with unhealed ulcers, limb amputation, impaired walking function, and very poor prognosis^1^. Although revascularization, including endovascular treatment and bypass surgery, has the most important role in treatment of CLTI, that is not indicated in 20–30% of affected patients. Furthermore, even when revascularization is performed, major amputation within three years is required in approximately 50% of those cases^2,3^. Cellular therapy, which can induce angiogenesis and improve ischemic tissue perfusion, is a promising approach for CLTI patients with limited therapeutic options. Several studies have suggested beneficial effects of cellular therapy for CLTI, with most of the investigated cases receiving a local muscular injection or intra-arterial administration as the cellular delivery route^4,5^. However, for highly frail CLTI patients with severely damaged ischemic limbs, such delivery methods are invasive and associated with risk for fatal complications including infection of the ischemic limb. In contrast, intravenous infusion of stem cells is a minimally invasive cellular delivery method and can be safely performed in CLTI patients, though a few have been reported in a CLTI animal model or patients.

Previously, we demonstrated the therapeutic effects and mechanisms of bone marrow-derived mesenchymal stem cells (MSCs) transplantation via intravenous administration for cerebrovascular ischemic disease^6–10^. When considering cell therapy for CLTI via intravenous delivery, inaccessibility to the ischemic limb is a major concern, because the lower limb is larger than the brain and hypoperfusion of a limb is quite severe in patients with CLTI. However, homing capacity of MSCs, which refers to migration of cells to injured or inflamed tissue, potentially overcome this problem and provide the therapeutic effects such as angiogenesis in an ischemic limb^11^. In this study, we hypothesized that intravenously infused MSCs induced angiogenesis and improved tissue perfusion through homing effect in a rat hindlimb ischemia model.

## Results

### MSC intravenous infusion increased tissue perfusion in ischemic limb

**Figure 1:**
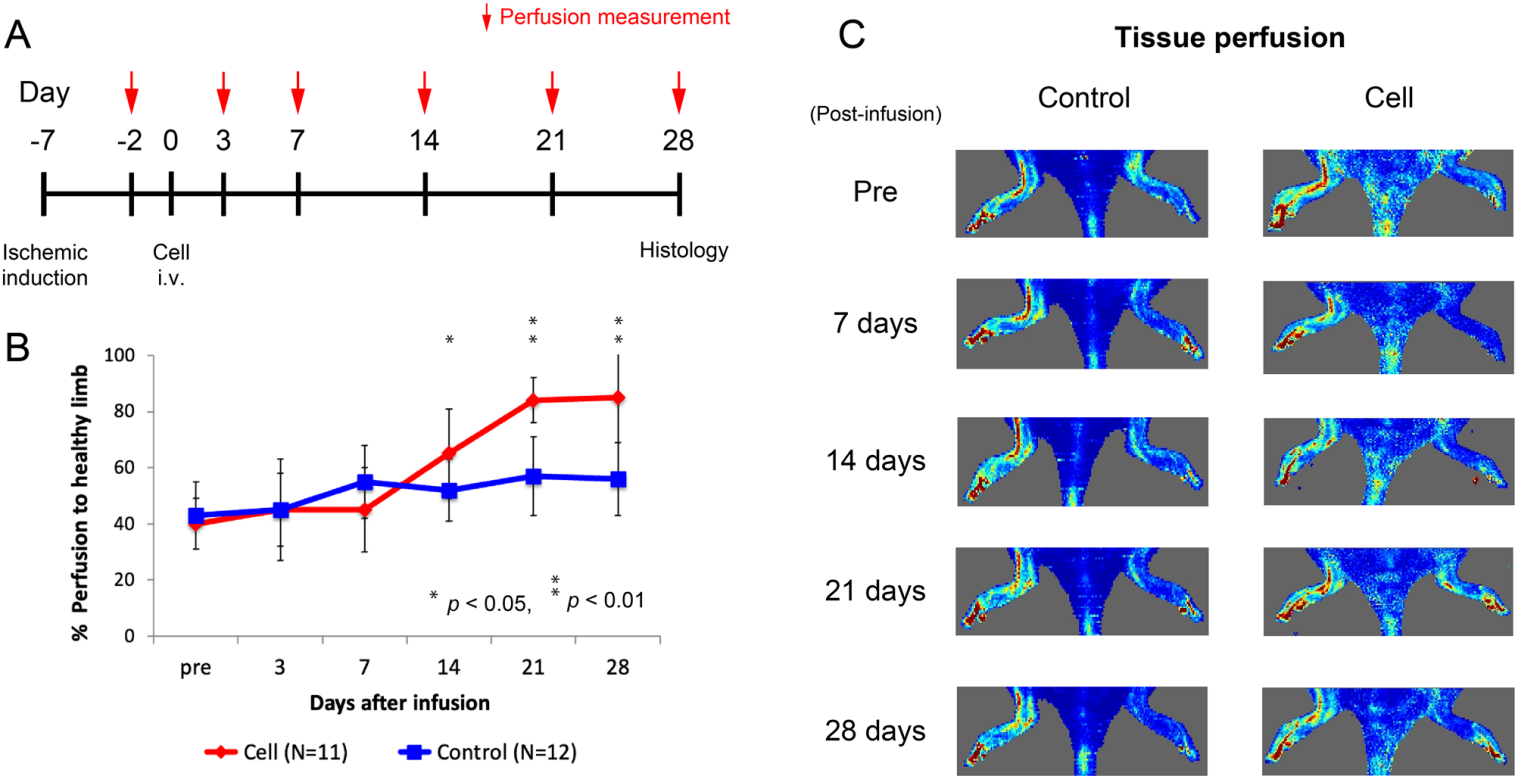
Tissue perfusion determined with laser Doppler. **(A)** Study timeline. **(B)** Ischemic hindlimb perfusion measurements after cell infusion. **(C)** Representative images of tissue perfusion in control and cell infusion groups. *p < 0.05, **p < 0.01.

Following intravenous infusion of 1 × 10^6^ MSCs (cell group, *n* = 11) or vehicle (control group, *n* = 12), tissue perfusion in the hindlimb was measured over the following 28 days (Fig. 1A), with representative serial perfusion images from each group shown in Fig. 1B. Prior to cell infusion, laser Doppler perfusion image (LDPI) index was not significantly different between the control and cell groups (43 ± 12% vs. 40 ± 9%, *p* = 0.58). From 7 to 21 days after cell infusion, tissue perfusion was improved in the cell group (Fig. 1C). The difference in LDPI index between the groups reached statistical significance at 14 days after cell infusion (52 ± 11% vs. 65 ± 16%, *p* = 0.03), and the improvement was sustained at 21 (57 ± 14% vs. 84 ± 8%, *p* < 0.001) and 28 (56 ± 13% vs. 85 ± 16%, *p* = 0.002) days.

### Intravenous MSC infusion induced angiogenesis

**Figure 2:**
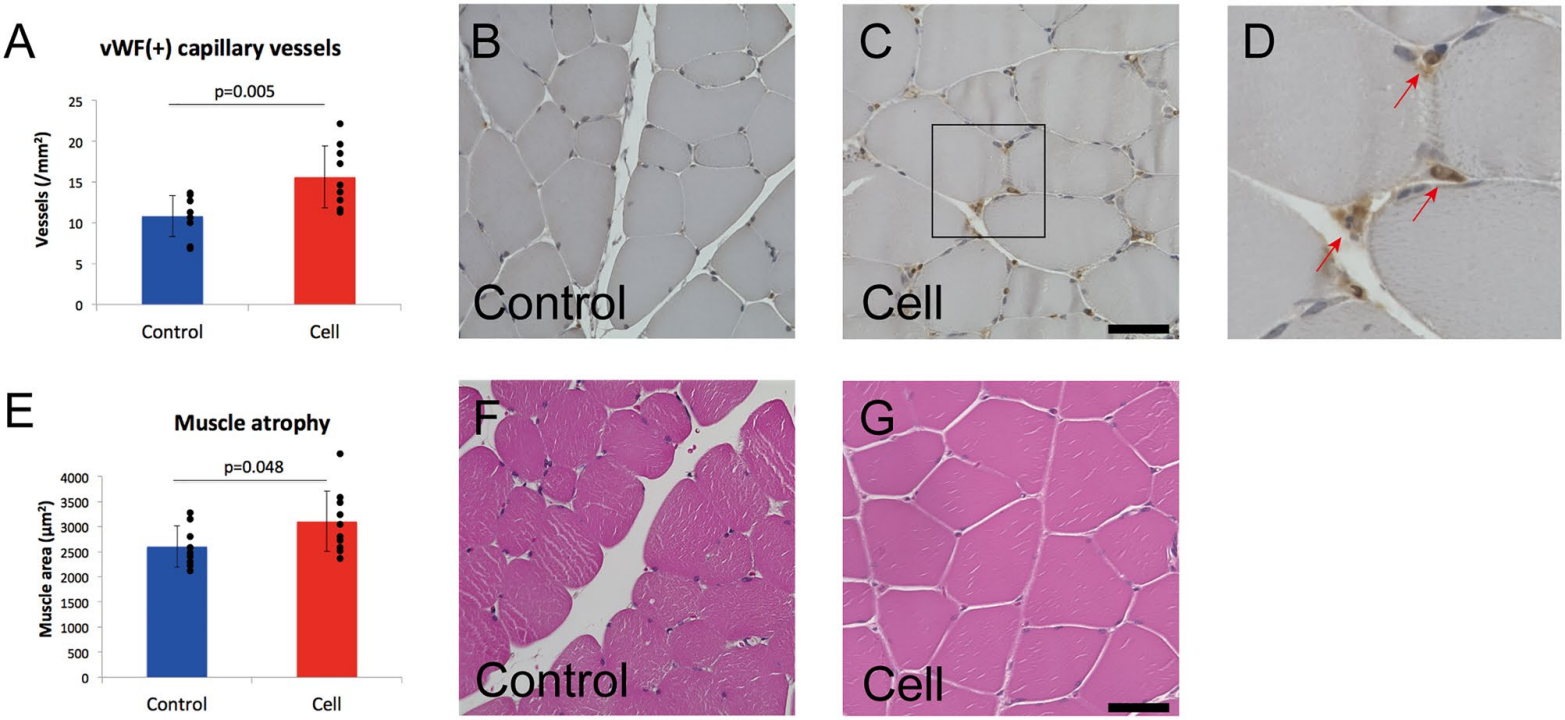
Histological assessment of ischemic hindlimb muscle. **(A)** Capillary vessel density. **(B,C)** Immunostaining of capillary vessels with anti-vWF (brown) and nuclei (blue). **(D)** Red arrows indicate capillary vessels. **(E)** Quantification of muscular atrophy. **(F,G)** Muscle fibers stained with hematoxylin and eosin. Scale bars in **(B)** to **(C)** indicate 50 μm, and in **(F,G)** indicate 100 μm.

Capillary numbers were evaluated on tissue cross-sections of adductor muscle at 28 days after cell infusion. As compared to the control, the number of capillary vessels was significantly increased in the cell group (108 ± 25/mm^2^ vs. 156 ± 38/mm^2^, *p* = 0.005) (Fig. 2A). Representative images of capillary staining are shown in Fig. 2B– D. In addition, the muscle fiber area in the cell group was increased as compared to that in the control group (2602 ± 409 μm^2^ vs. 3102 ± 601 μm^2^, *p* = 0.048), suggesting that MSC infusion attenuated muscular atrophy caused by ischemia (Fig. 2E). Representative images of hematoxylin and eosin staining are shown in Fig. 2F,G.

### Infused green fluorescent protein (GFP)-MSCs detected in ischemic lesions

**Figure 3:**
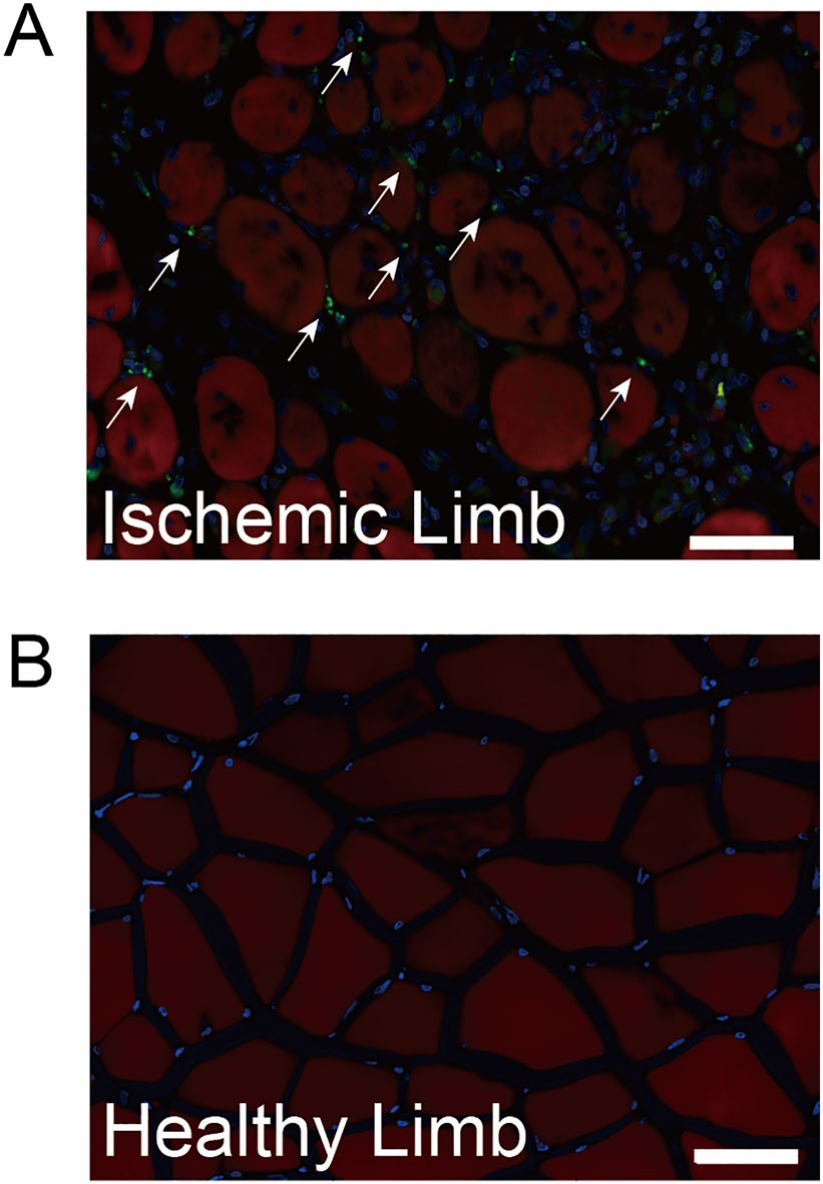
Histological assessment of rat ischemic limb tissues 1 day after GFP-MSC infusion. **(A)** In the cell group, infused GFP-MSCs (green) were detected in the ischemic hindlimb after counterstaining with phalloidin (red) and DAPI (blue). **(B)** In the control group, no green cells were found. Scale bars indicate 50 μm.

The distribution of GFP-MSCs (green) was histologically assessed at one day after cell infusion. Infused GFP-MSCs were detected in the ischemic hindlimbs (Fig. 3A), while none were detected in the contralateral healthy hindlimb (Fig. 3B). Similarly, no green cells were found in the control group.

### MSC infusion upregulated vascular endothelial growth factor (VEGF) gene expression in ischemic tissue

**Figure 4:**
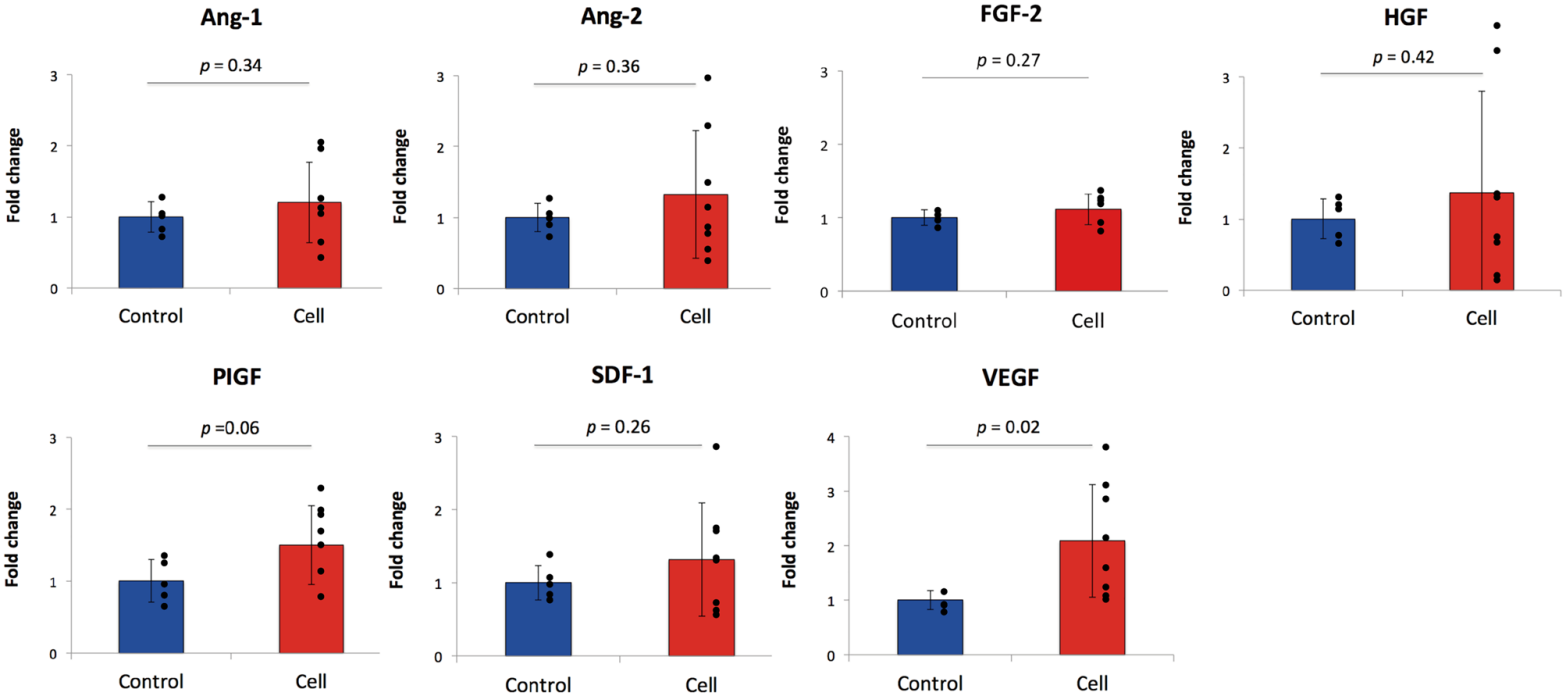
qPCR results of rat muscle tissues showing angiogenic factor gene expressions in the control and cell infusion groups.

Gene expression of angiogenic cytokines was analyzed in ischemic hindlimb muscles harvested three days after MSC infusion. While gene expression of most cytokines was not different between the groups, gene expression of VEGF was significantly increased in the cell group (*p* = 0.02) and that of placenta growth factor (PIGF) showed a tendency to increase (*p* = 0.06) (Fig. 4).

## Discussion

In this report, we demonstrated that intravenous infusion of bone-marrow derived MSCs increased tissue perfusion in a rat hindlimb ischemia model. Histological results showed induction of angiogenesis and attenuation of muscular atrophy in ischemic hindlimb muscles treated with MSC infusion. Gene expression of VEGF was significantly upregulated in ischemic hindlimb muscles treated with MSC infusion and there was a tendency for upregulation of PIGF gene expression. Furthermore, infused GFP-MSCs were detected in the ischemic limbs, suggesting an MSC homing effect. We concluded that intravenous infusion of bone-marrow derived MSCs improved tissue perfusion in ischemic hindlimbs through angiogenesis, which was suggested to be induced by migration of MSCs to ischemic tissue.

In this study, we examined the therapeutic effects of intravenous infusion of MSCs in a rat limb ischemia model. Although beneficial effects of intravenous cell therapy for chronic stroke or ischemic cardiomyopathy have been observed in clinical trials, the underlying mechanisms remain unclear^12,13^. Our results showed accumulation of intravenously infused MSCs in ischemic limbs, while that was not seen in healthy limbs. Those are consistent with findings of a previous study, in which intravenously administered MSCs were detected only in the ischemic side of brain tissue examined in a rat stroke model^7^. Accumulation of MSCs in ischemic organs suggests that they have a homing effect, which may explain the therapeutic mechanism of intravenous infusion of MSCs for ischemic disorders. Homing is the process by which systemically circulating cells are recruited to impaired or inflamed tissue via the endothelial vasculature, and induced by expression of a receptor or ligand to facilitate trafficking, adhesion, and infiltration of MSCs into injured tissue^11^. In ischemic tissue, hypoxia-inducible factor-1 (HIF-1) upregulates stromal cell-derived factor-1 (SDF-1) and its receptor CXCR4, leading to mobilization and migration of stem cells^14,15^, a possible mechanism by which intravenously infused MSCs were found accumulated in ischemic limbs of the present rat model. On the other hand, other researchers have insisted that the therapeutic effects of intravenously infused MSCs for an ischemic disorder were derived from systemic effects caused by accumulation of infused cells in other organs. Lee et al.^16^ reported that intravenously infused MSCs embolized in lung secreted anti-inflammatory protein, leading to reduction of infarct size in a mouse myocardial infarction model. Luger et al.^17^ also showed that intravenously infused MSCs accumulated in the spleen, which mediated anti-inflammatory activity and attenuated deterioration of left ventricular function in a mouse myocardial infarction model. Similarly, for CLTI, accumulation of intravenously infused MSCs in other organs may improve ischemic symptoms through secretion of angiogenic or anti-inflammatory factors. Furthermore, some researchers^18^ have proposed the effectiveness of MSC injection via intravenous regional limb perfusion in an equine tendon injury model, which may be a reasonable option for treatment of limb ischemia in human patients. Although intravenous cell therapy is an attractive therapeutic option for high risk CLTI patients, elucidation of the precise mechanism of such therapy for ischemic diseases remains challenging.

In the present study, gene expression of angiogenic cytokines in ischemic hindlimb muscles was analyzed, with upregulation of VEGF and a tendency for upregulation of PIGF shown. VEGF has been recognized to have a pivotal role in angiogenesis, though clinical trials of gene therapy utilizing VEGF have failed to show convincing evidence^19–21^. PIGF is a growth factor that belongs to the VEGF family and activates Flk1, a VEGF receptor, resulting in strengthened angiogenesis induced by VEGF via cross-talk mechanisms^22^. Autiero et al.^23^ showed that administration of PIGF and VEGF together induced angiogenesis more effectively than VEGF alone in a rat myocardial infarction model. Results of another study indicated that PIGF induced development of larger diameter arterioles with greater functional maturity, while VEGF induced capillaries that were fragile and functionally immature^24^. Therefore, intravenous infusion of bone-marrow derived MSCs, which leads to simultaneous upregulation of VEGF and PIGF in an ischemic limb, potentially induces angiogenesis more effectively and sufficiently improves tissue perfusion.

## Conclusions

Intravenously infused bone-marrow derived MSCs improved tissue perfusion in ischemic hindlimbs in a rat model. Angiogenesis was induced by cytokines including VEGF and PIGF, and accumulation of infused MSCs in ischemic limbs may explain the angiogenesis induced by intravenous infusion of MSCs. Our findings suggested that intravenous infusion of MSCs would be a promising cell delivery method for treatment of CLTI.

## Materials and methods

All animal protocols were approved by the Animal Experimentation Committee of Osaka University and the Animal Care and Use Committee of Sapporo Medical University, and the Committee for Security of Recombinant DNA Experiments of Sapporo Medical University, and performed according to the Guidelines for Animal Experiments of the each institution. In addition, all animal protocols were conducted in accordance with ARRIVE guidelines.

### Cell preparation

The method used for MSC culturing was previously described^25^. Briefly, bone marrow was obtained from femoral bones of adult Sprague–Dawley rats or GFP-expressing Sprague–Dawley rats (W-Tg [CAG-GFP]184Ys), then diluted with 15 ml DMEM (Sigma) supplement, 10% heat-inactivated fetal bovine serum (Thermo Fisher Scientific Inc.), 2 mM 1-glutamine (Sigma), 100 U/ml penicillin, and 0.1 mg/ml streptomycin (Thermo Fisher Scientific Inc.), and incubated for 3 days in an atmosphere containing 5% CO_2_ at 37 °C. Following confluence, adherent cells were detached with trypsin-ethylenediaminetetraacetic acid solution (Sigma) and subcultured at 1 × 10^4^ cells/ml. In our previous study, morphological features of the bone marrow-derived MSCs, including characteristically flattened and spindle-shaped cells that appeared as plastic adherent cells, were reported^26^. We also performed a phenotypic analysis of surface antigens, which revealed cluster of differentiation (CD)45−, CD73+, CD90+, and CD106− on MSCs, and confirmed that MSCs gave rise to mesenchymal derivatives, including osteocytes, adipocytes, and chondrocytes^27,28^. For the present study, MSCs were used after three passages.

### Rat hindlimb ischemia model and blood perfusion measurement

Female Sprague–Dawley rats (10 weeks old) were purchased from SLC Japan (Shizuoka, Japan). A hindlimb ischemia model was established by removing the left femoral artery and vein, as previously described^29^. At 5 days after ligation, tissue perfusion was assessed using a LDPI analyzer (Moor Instruments, Axminster, UK), then rats with an LPDI index > 70% were excluded. LDPI index was calculated as the ratio of perfusion in the ischemic hindlimb to that in the healthy hindlimb. At 7 days after ligation, 1 × 10^6^ MSCs in 1 ml of DMEM (cell group) or 1 ml of vehicle (fresh DMEM, no cells: control group) were infused intravenously from a 24-gauge catheter inserted into the right internal jugular vein. With the tip of catheter inserted into the heart, MSCs or vehicle were slowly administered. At 7, 14, 21, and 28 days after cell infusion, tissue perfusion was determined with the LDPI analyzer and the LDPI index calculated. For this study, we used female rats with relatively mild characteristics because of the frequent invasive procedures, though we considered that it was a study limitation. On the other hand, a recent paper^30^ noted that firm and evidence-based guidance for the design of including both sexes in peripheral artery disease-related animal studies was not possible because sex differences were less described in patients with peripheral artery disease. For immunosuppression, cyclosporine A (10 mg/kg/day, i.p.) was administered daily from 48 h before cell infusion. Since the experiments in this study were not autologous transplantation, cyclosporine A was used to avoid a potential immune reaction^6,7,25,31–40^.

### Histological analysis

Rats were euthanized with CO_2_ at 28 days after cell infusion. The adductor muscle was dissected, then fixed with formalin and embedded in paraffin, and cut into 5-µm sections using a microtome for histological analysis. For assessment of capillary vessels, ischemic muscle sections were stained with sheep polyclonal anti-von Willebrand factor (vWF) antibody (AB7356, 1:50; Millipore). Furthermore, sections were stained with hematoxylin for assessment of muscle atrophy based on average fiber size, calculated as the ratio of muscle area to number of muscle fibers. For detection of infused MSCs in the ischemic limb, rats were euthanized at one day after GFP-MSC infusion, then frozen sections were stained with DAPI and phalloidin (A34055, 1:100; Invitrogen). Rats treated with the vehicle were examined as a control. Histological measurements were performed in five randomly selected fields of each tissue section. Obtained images were examined using an optical microscope (Keyence, Osaka, Japan).

### Real-time quantitative polymerase chain reaction (RT-PCR)

Gene expression of angiogenic factors in muscle samples was determined using a RT-PCR assay. Adductor muscles were harvested five days after cell transplantation or vehicle injection, then the samples were immersed in RNA (Invitrogen). Total RNA was isolated using an RNeasy Kit (Qiagen, Hilden, Germany) and reverse-transcribed with an Omniscript Reverse Transcriptase kit (Qiagen). RT-PCR was performed using TaqMan Gene Expression Assay Master Mix (Applied Biosystems, California, US) with the 7500 Fast Real-Time PCR System (Applied Biosystems). Primers used in this study were as follows: angiopoietin-1 (Ang-1) (Assay ID: Rn00585552_m1), Ang-2 (Assay ID: Rn01756774_m1), fibroblast growth factor-2 (FGF-2) (Assay ID: Rn00570809_m1), GAPDH (Assay ID: Rn01775763_g1), hepatocyte growth factor (HGF), (Assay ID: Rn00566673_m1), PIGF (Assay ID: Rn01472372_m1), SDF-1 (Assay ID: Rn00573522_s1), and VEGF (Assay ID: Rn01511601_m1).

### Statistical analysis

All continuous variables are summarized as mean ± standard deviation and were compared using Welch’s *t*-test. *P*-values are two-sided and those < 0.05 were considered to be statistically significant. All statistical analyses were performed using JMP Pro14 (SAS Institute, Cary, NC).

## Data Availability

Datasets for this study are available from the corresponding author upon reasonable request.
